# Leukemia cutis simulating drug reaction with eosinophilia and systemic symptoms following beta-lactam antibiotic use

**DOI:** 10.1016/j.jdcr.2024.04.002

**Published:** 2024-04-16

**Authors:** Jaclyn Abraham, Navid Farahbakhsh, Kiran Motaparthi

**Affiliations:** a University of South Florida Morsani College of Medicine, Tampa, Florida; b Department of Dermatology, University of Florida College of Medicine, Gainesville, Florida

**Keywords:** acute monocytic leukemia, acute myeloid leukemia, antibiotics, DRESS, drug reaction, leukemia cutis, morbilliform

## Introduction

Leukemia cutis (LC) is an extramedullary manifestation of leukemia that presents as discernible cutaneous lesions composed of neoplastic leukocytes.^1^^,^^2^ It is estimated that 10% to 15% of patients with acute myeloid leukemia (AML) develop LC, with varying frequency based on the subtype of AML.^1^ A wide range of morphological findings are associated with LC, including erythematous, red-brown, or violaceous papules, plaques, nodules, or tumors that can ulcerate or become bullous.^3^ Lesions often present on the head, neck, and trunk, with an affinity for sites of prior or concurrent inflammation.^1^ We describe an atypical presentation of LC which fulfilled RegiSCAR criteria for drug eruption with eosinophilia and systemic symptoms (DRESS) following the recent completion of a beta-lactam antibiotic course.

## Case report

A 41-year-old woman was evaluated by the dermatology inpatient consult service for a 2-day history of a pruritic eruption involving the face, neck, trunk, and bilateral upper extremities. The patient was febrile (up to 38.6 °C) and reported facial swelling with a sore throat. Four days prior to hospital admission, the patient completed a 1-week prophylactic course of cefalexin following a double mastectomy for recently diagnosed stage IA breast cancer.

**Fig 1:**
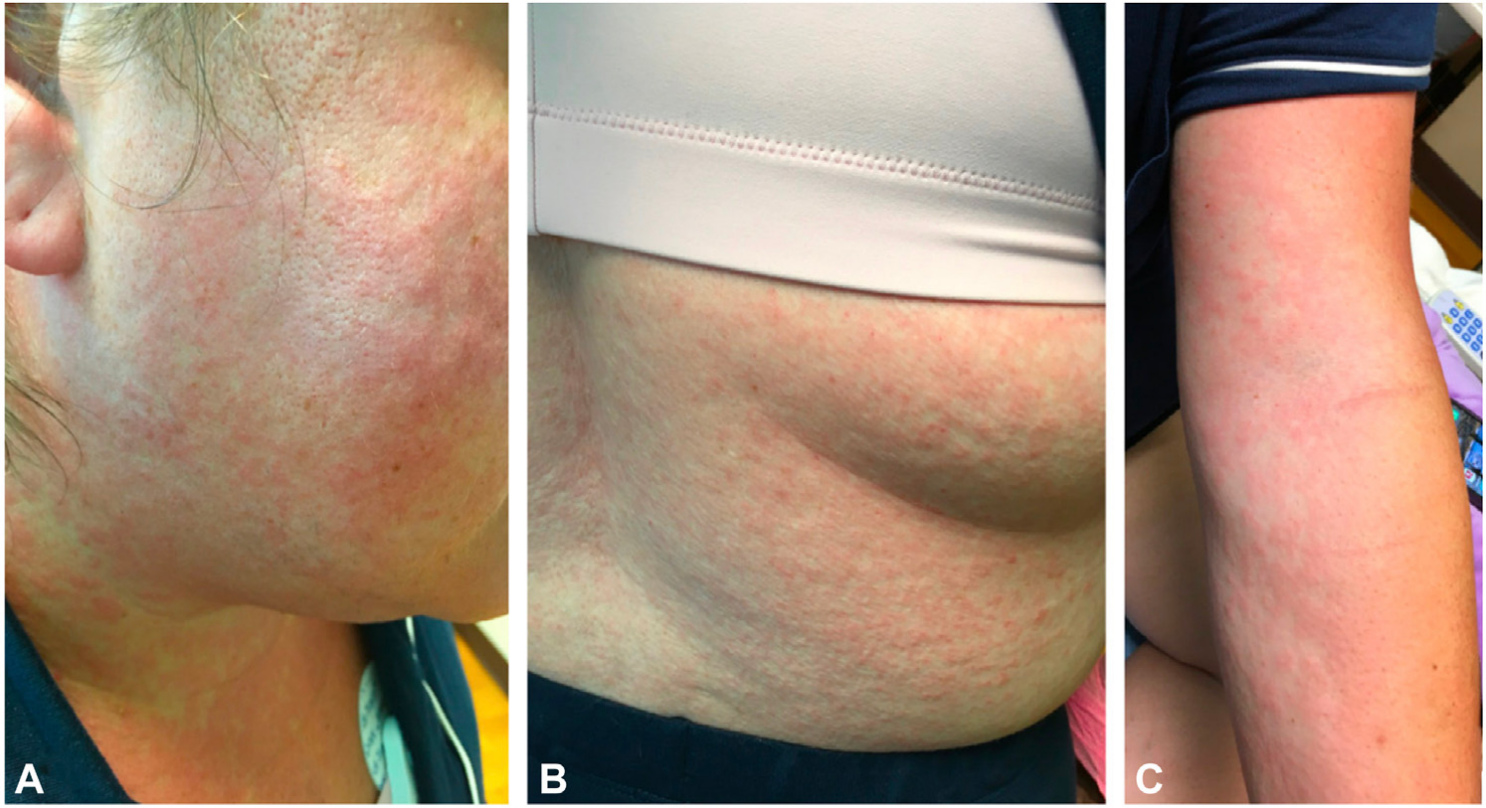
Diffuse erythematous macules and papules symmetrically distributed on the face and neck (**A**), flank (**B**), and forearm (**C**).

Numerous erythematous macules and papules involving the face, neck, trunk, upper extremities, and proximal thighs were present (Fig 1). Physical examination was also significant for cervical, axillary, and inguinal lymphadenopathy and facial edema.

Initial laboratory studies showed leukocytosis (white blood cell count of 66.1 × 10^3^/μL), eosinophilia (absolute eosinophil count of 990 cells/μL), monocytosis (17%), elevated creatinine (1.31 mg/dL), and mild transaminitis (aspartate aminotransferase 57 U/L, alanine aminotransferase 92 U/L). Serological testing for Epstein-Barr virus, cytomegalovirus, human immunodeficiency virus, *Treponema* *palladium* IgG, and hepatitis B and C were negative. A peripheral blood smear showed atypical lymphocytes.

Based on the clinical and laboratory findings, the differential diagnosis included DRESS, favored based on the patient’s symptoms and laboratory findings with a latency of 1 week following beta-lactam administration. The patient had a calculated RegiSCAR score of 7 at the time of presentation, indicating a “definite case” of DRESS (Table I).^3^^,^^4^ However, the patient’s hyperleukocytosis prompted further workup to rule out other disease etiologies, including an underlying hematologic malignancy.

**Table I:** Diagnosis of drug eruption with eosinophilia and systemic symptoms based on RegiSCAR criteria^3^^,^^4^

Features	No	Yes	Unknown
Fever (>38.5 °C)	−1	**1**	−1
Lymphadenopathy (2 or more sites, >1 cm)	0	**1**	0
Atypical lymphocytes	0	**1**	0
Eosinophilia	0	−	0
0.7-1.499 × 10^9^/L		**1**	
≥1.5 × 10^9^/L		2	
Skin rash extent >50%	0	**1**	0
At least 2 of: edema, purpura, infiltration, scaling	**0**	1	0
Biopsy suggesting DRESS	−1	1	**0**
Internal organ involved	0	−	0
One		1	
Two or more		**2**	
Resolution in >15 days	−1	0	**−1**
Alternative diagnoses excluded (by ≥3 biological investigations)	0	**1**	0

Final RegiSCAR group score interpretation: <2, no case; 2 to 3, possible case; 4 to 5, probable case; >5, definite case; bolded values represent features specific to the case presented (total = 7 points).

*DRESS*, Drug eruption with eosinophilia and systemic symptoms.

**Fig 2:**
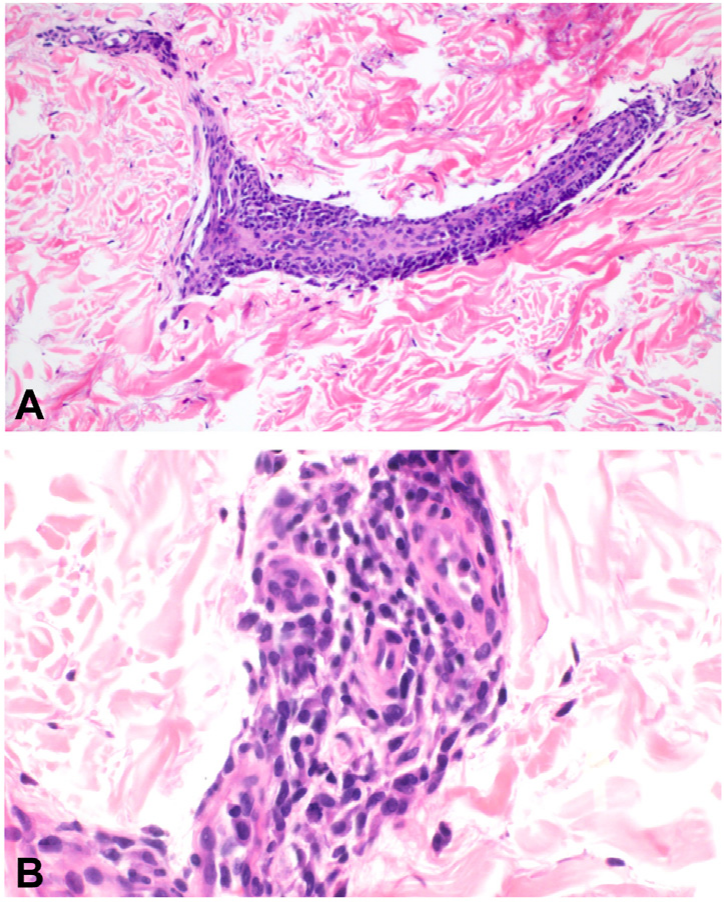
Dermal and subcutaneous infiltrate of perivascular medium-sized mononuclear cells (**A**) with enlarged hyperchromatic nuclei consistent with blasts (**B**) (**A** and **B,** Hematoxylin-eosin stain; original magnification: **A** and **B,** 400× magnification).

A punch biopsy was performed. Hematoxylin-eosin–stained sections revealed a dermal and subcutaneous infiltrate of perivascular medium-sized mononuclear cells with enlarged hyperchromatic nuclei consistent with blasts (Fig 2).

Immunohistochemical staining revealed CD4 and CD56 positive cells; medium-sized blasts expressed CD43, lysozyme, and CD68. Markers for CD34, CD117, CD123, and myeloperoxidase were negative. Bone marrow biopsy and flow cytometry of the bone marrow and blood matched the immunohistochemical profile from the skin, which was consistent with acute monocytic leukemia (AMoL). Lymph node biopsy demonstrated myeloid sarcoma. Based on these results, the diagnosis of LC secondary to AMoL was confirmed. Given that our patient had 2 new simultaneous, but unrelated malignancies, the oncology team prioritized the treatment for AMoL. The patient’s rash and systemic symptoms slowly improved following chemotherapy.

## Discussion

This case of LC simulating DRESS underscores the high index of clinical suspicion required to diagnose LC, particularly in patients without a known hematologic malignancy. Cutaneous involvement as the presenting feature of leukemia is rare, as most patients who present with LC will already have an established diagnosis of leukemia.^5^ Moreover, our patient presented with an acute, pruritic morbilliform (exanthematous) eruption and systemic symptoms, which contrasts typical presentation of LC. LC usually presents with smooth, erythematous to violaceous papules, plaques, or nodules with a localized or disseminated distribution.^5^ Very few studies have described cases of LC simulating inflammatory dermatoses.^6^^,^^7^ For example, Donaldson et al reported a case of LC presenting as erythroderma shortly after induction chemotherapy for AML.^6^

In addition to our patient’s atypical cutaneous presentation, the temporal relationship between the patient’s symptom onset and recent beta-lactam antibiotic use posed an additional diagnostic challenge. While DRESS typically presents 2 to 8 weeks after administration of the inciting drug,^3^ recent studies have suggested there is a significantly shorter latency period for beta-lactam antibiotics compared to other drug classes. The median latency period for patients with suspected beta-lactam-induced DRESS was 4.5 days compared to 16 days for vancomycin-associated DRESS.^3^

Of note, our patient presented with markedly elevated leukocytosis (66 × 10^3^/μL). Although DRESS can present with leukemoid reactions including white blood cell counts upward of 50 × 10^3^/μL, hyperleukocytosis at the time of an AML diagnosis is a rare but notable feature that portends a poor prognosis.^8^ This significant hematologic abnormality prompted further workup, which led to the prompt diagnosis of LC secondary to AMoL in our patient.

For an acute exanthematous eruption with fever and lymphadenopathy, the differential diagnosis includes mononucleosis with recent beta-lactam use, acute retroviral syndrome due to human immunodeficiency virus, secondary syphilis, DRESS, and angioimmunoblastic T-cell lymphoma (Table II). Although rare, physicians should also consider LC in patients presenting with morbilliform rash, fever, and lymphadenopathy.

**Table II:** Differential diagnosis for acute exanthematous eruption with fever and lymphadenopathy

	LC	DRESS	Mononucleosis	ARS	Secondary syphilis	AITL
Onset	Varies	Two-8 wk following drug exposure	Day ∼4 of illness; recent beta-lactam antibiotic use	Three-6 wk following HIV exposure	Three-10 wk following chancre	Varies
Distribution of lesions	Face, trunk, extremities	Face, upper trunk, extremities	Trunk and proximal extremities	Widespread	Widespread	Widespread
Additional features	Malaise, headache, arthralgias, hepatosplenomegaly	Facial edema, pruritus	Malaise, pharyngitis, splenomegaly	Malaise, myalgias, pharyngitis, orogenital ulcerations	Flu-like illness, weight loss, pharyngitis, mucosal lesions, alopecia, hepato-splenomegaly	Pruritus, weight loss, night sweats, hepatosplenomegaly
Diagnosis	Skin biopsy	RegiSCAR criteria	Monospot test	HIV serological assays	*T palladium* IgG	Lymph node biopsy
Treatment	Chemotherapy for underlying malignancy	Withdrawal of offending agent	Supportive	Antiretroviral therapy (ART)	Intramuscular benzathine penicillin G	Chemotherapy

*AITL*, Angioimmunoblastic T-cell lymphoma; *ARS*, acute retroviral syndrome; *DRESS*, drug eruption with eosinophilia and systemic symptoms; *LC*, leukemia cutis.

LC often indicates advanced disease and a poorer prognosis, since 90% of patients will have additional sites of extramedullary disease involvement.^1^^,^^2^^,^^10^ Diagnosis is made based on clinical presentation, cytology, and the immunophenotype of malignant cells.^1^ Peripheral blood findings and bone marrow biopsy can also help establish the diagnosis.^1^ Remission of the hematologic abnormalities and partial to complete resolution of the cutaneous infiltrates typically follows chemotherapy.^5^^,^^7^

Although the RegiSCAR criteria are widely used to clinically diagnose DRESS, these were initially established for research studies but were not validated for use in the clinical setting.^4^ Limitations include lack of specificity for certain criteria, including hematologic abnormalities, organ involvement, and skin biopsy findings. For example, the presence of atypical lymphocytes may also be observed in viral infections; conversely, patients with DRESS and pancytopenia may not develop atypical lymphocytes or eosinophilia.^4^ RegiSCAR also requires the exclusion of at least 3 of the following: hepatitis A/B/C, *Chlamydia* or *Mycoplasma* infection, antinuclear antibody, and blood culture. However, RegiSCAR does not require testing for other viral infections that may be clinically indiscernible from DRESS, such as Epstein-Barr virus or cytomegalovirus infection.^4^ Therefore, we aim to raise awareness of these important limitations when utilizing diagnostic criteria in any clinical scenario where DRESS enters the differential diagnosis.

## Conflicts of interest

None disclosed.

## References

[1] Cho-Vega J.H., Medeiros L.J., Prieto V.G., Vega F. (2008). Leukemia cutis. Am J Clin Pathol.

[2] Ratnam K.V., Khor C.J., Su W.P. (1994). Leukemia cutis. Dermatol Clin.

[3] Waldron J.L., James F., Vogrin S. (2023). A shorter time to drug reaction with eosinophilia and systemic symptoms (DRESS): redefining beta-lactam-associated DRESS. Clin Infect Dis.

[4] Sibbald C., Shear N.H., Verstegen R.H.J. (2023). Flaws and limitations of classification criteria for drug reaction with eosinophilia and systemic symptoms. J Allergy Clin Immunol Pract.

[5] Parsi M., Go M.S., Ahmed A. (2023).

[6] Martínez-Escanamé M., Zuriel D., Tee S.I., Fried I., Massone C., Cerroni L. (2013). Cutaneous infiltrates of acute myelogenous leukemia simulating inflammatory dermatoses. Am J Dermatopathol.

[7] Donaldson M., Ebia M.I., Owen J.L., Choi J.N. (2019). Rare case of leukemia cutis presenting as erythroderma in a patient with acute myeloid leukemia. JAAD Case Rep.

[8] Daver N., Kantarjian H., Marcucci G. (2015). Clinical characteristics and outcomes in patients with acute promyelocytic leukaemia and hyperleucocytosis. Br J Haematol.

[9] Bolognia J., Jorizzo J.L., Schaffer J.V. (2012).

[10] Wang C.X., Pusic I., Anadkat M.J. (2019). Association of leukemia cutis with survival in acute myeloid leukemia. JAMA Dermatology.

